# Development and Validation of a Trigger Tool for Identifying Drug-Related Emergency Department Visits

**DOI:** 10.3390/ijerph18168572

**Published:** 2021-08-13

**Authors:** Sung-Hee Hwang, Young-Mi Ah, Kwang-Hee Jun, Jae-Woo Jung, Min-Gyu Kang, Hye-Kyung Park, Eui-Kyung Lee, Hye-Kyung Park, Jee-Eun Chung, Sang-Heon Kim, Ju-Yeun Lee

**Affiliations:** 1 College of Pharmacy and Institute of Pharmaceutical Science and Technology, Hanyang University, Ansan 15588, Korea; shhwang8@hanyang.ac.kr (S.-H.H.); jechung@hanyang.ac.kr (J.-E.C.); 2 College of Pharmacy, Yeungnam University, Gyeongsan 38541, Korea; ymah@ynu.ac.kr; 3 Institute of Pharmaceutical Sciences, College of Pharmacy and Research, Seoul National University, Seoul 08826, Korea; culturevo@snu.ac.kr; 4 Department of Internal Medicine, Chung-Ang University College of Medicine, Seoul 06974, Korea; jwjung@cau.ac.kr; 5 Department of Internal Medicine, Chungbuk National University Hospital, Cheongju 28644, Korea; irreversibly@gmail.com; 6 Department of Internal Medicine, Pusan National University College of Medicine, Busan 50612, Korea; parkhk@pusan.ac.kr; 7 School of Pharmacy, Sungkyunkwan University, Suwon 16419, Korea; ekyung@skku.edu (E.-K.L.); phk94@skku.edu (H.-K.P.); 8 Department of Internal Medicine, Hanyang University College of Medicine, Seoul 04763, Korea

**Keywords:** adverse drug events, safety, emergency department, trigger tool, medication safety

## Abstract

There are various trigger tools for detecting adverse drug events (ADEs), however, a drug-related emergency department (ED) visit trigger tool (DrEDTT) has not yet been developed. We aimed to develop and validate a DrEDTT with a multi-center cohort. In this cross-sectional study, we developed the DrEDTT consisting of 28 triggers through a comprehensive literature review and three phase expert group discussion. Next, we evaluated the performance of the DrEDTT by applying it to relevant medical records retrieved from four hospitals from January 2016 to June 2016. Two experts performed an in-depth chart review of a 25% of random sample of trigger flagged and unflagged ED visits and a true ADE was determined through causality assessment. Among 66,564 patients who visited the ED for reasons other than traffic accident and trauma during the study period, at least one trigger was found in 21,268 (32.0%) patients. A total of 959 true ADE cases (5.8%) were identified from a randomly selected 25% of ED visit cases. The overall positive predictive value was 14.0% (range: 8.3–66.7%). Sensitivity and specificity of DrEDTT were 77.7% and 70.4%, respectively. In conclusion, this newly developed trigger tool might be helpful to detect ADE-related ED visits.

## 1. Introduction

Adverse drug events (ADEs), defined as harm resulting from an appropriate or inappropriate use of drugs [1], represent an important aspect of quality of care. It was reported that 2.3–21.3% of adult in-patients [2] and 0.2–34.7% of outpatients [3] experience ADEs [3]. Given the widespread recognition that ADEs are both common and costly, many governments and hospitals are seeking multifaceted approaches to reduce drug-related harm in order to provide better quality care at lower costs. To establish effective preventive measures for ADEs, the prevalence and the type of ADEs should be evaluated.

Evaluating and understanding ADEs and establishing a policy to prevent them remain greater challenges in outpatient settings than inpatient settings, owing to the difficulty of obtaining nationally representative surveillance data on outpatients [4,5]. Determining the prevalence of emergency department (ED) visits for drug-related problems can be an alternative method and may be an effective community ADE surveillance strategy in addition to spontaneous reports or epidemiologic study data obtained from outpatient clinics. ED-based ADE studies are also meaningful, in that they can assess the prevalence of serious ADEs requiring additional treatments [6].

The concept of a trigger tool for detecting potential ADEs recorded in medical records was first introduced by Jick in 1974 and refined by Classen et al. in 2003 [7]. Although limited studies validated the trigger tool method compared with prospective criterion standard and have not always shown encouraging results [8], the trigger tool method appears to be a more effective and labor-efficient method for identifying ADEs compared to the conventional chart review method in a retrospective study [9]. Trigger tools developed by the Institute for Healthcare Improvement (IHI) have been commonly used and many studies have evaluated the prevalence of ADEs using trigger tools in various settings [10].

However, existing trigger tools are more focused on inpatients than outpatients, and a specific trigger tool for drug-related ED visits has not yet been developed. Griffey et al. developed the emergency department trigger tool (EDTT) [11] to identify ADEs caused by medical interventions in the ED setting. The previously developed trigger tools for ambulatory settings [12,13,14] could not be applied in the ED setting directly as previous laboratory results or medication history was often unavailable. A study that evaluated the performance of a previously developed trigger for detecting drug-related ED visits showed negative results [8].

Therefore, we aimed to develop a specific trigger tool to identify drug-related ED visits and to validate them using data retrieved from four university hospitals.

## 2. Methods

### 2.1. Study Setting

This cross-sectional study was conducted in two stages. First, we developed a trigger tool to identify drug-related ED visits. In the second stage, we evaluated its performance of the tool by applying it to relevant medical records retrieved from four hospitals. The study protocol was approved by the institutional review boards of Hanyang University Hospital (2016-07-042), Chungbuk National University Hospital (CBNUH 2015-06-018), Chung-Ang University Hospital (C2015174), and Pusan National University Hospital (H-1608-004-044). The two hospitals were located in the capital of the country, and the other two hospitals were located in different provinces. The ED volume of each hospital was approximately 3750 (Chung-Ang University Hospital), 3400 (Chungbuk National University Hospital), 3100 (Hanyang University Hospital), and 2450 (Pusan National University Hospital) encounters per month. Even though the four hospitals are different in location and size, they all operate as Regional Pharmacovigilance Centres authorized by the Korea Institute of Drug Safety and Risk Management (KIDS), and were designated as regional emergency medical centers by the Ministry of Health and Welfare, Korea.

### 2.2. ADE Identification and Classification

As this study was conducted with the retrospective chart review using anonymized data, a waiver of participants’ informed consent was approved by local IRBs. All methods in the study were performed in accordance with relevant guidelines and regulations.

We included ADE cases with higher-than-possible causality after assessing causality based on the World Health Organization (WHO)-Uppsala Monitoring Centre (UMC) causality criteria [1].

We classified ‘preventable’ ADEs by using the criteria developed by Hallas et al. [15]: taking the wrong drug, taking a sub- or supra-therapeutic dose, and non-adherence. ADEs due to medications taken in the ED and intentional overdose or drug intoxication with suicidal intent were excluded.

The severity of ADEs was categorized based on the modified National Coordinating Council for Medication Error Reporting and Prevention index (NCC-MERP) [16]: serious, if the event required hospitalization or caused death; moderate, if the event required a change in medical management; and mild, if the event required no change in management.

### 2.3. Drug-Related ED Visit Trigger Tool Development

**Figure 1:**
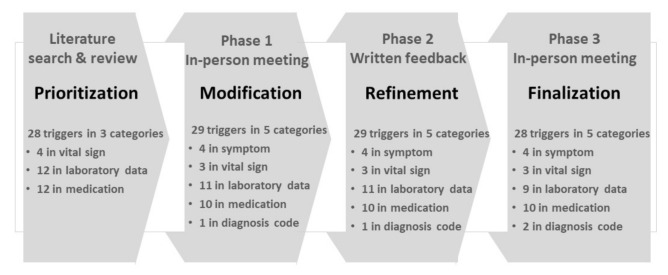
Process of developing the drug-related ED visit trigger tool (DrEDTT).

We developed the drug-related ED visit trigger tool (DrEDTT) in four steps (Figure 1). In the Prioritization phase, we selected the major types of ADEs that had been identified as drug-related problems leading to an ED visit from previous studies [5,17,18,19,20,21]. We generated the candidate trigger lists for each major type of ADE by using a previously developed ambulatory care-based trigger tool [7,12,13,14,15,16,17,18,19,20,21] and prioritized the category and individual triggers considering the following factors: availability of laboratory data and standardized information intended for the National Emergency Department Information System (NEDIS) [22] operated by the government, such as chief complaints, diagnosis, and vital signs as well as incomplete information on medical and medication history (prioritization phase). After Prioritization was complete, we further modified and finalized the DrEDTT based on a three-phase group discussion of experts (four internal medicine physicians in charge of regional pharmacovigilance center and one clinical pharmacist). Phase 1 (Modification) was performed through in-person group discussion where the experts reviewed the prioritized categories and triggers suggested from the literature review: two categories (symptom and diagnostic code) were added, and four triggers were removed. In Phase 2 (Refinement), we adjusted the cut-off values of laboratory triggers and specific medications included in medication triggers through online written feedback. The experts from different hospitals gave their opinion after pilot application of triggers and with consideration of hospital’s practice pattern. In Phase 3 (Finalization), the DrEDTT was further modified and finalized in a group meeting: laboratory triggers of renal failure were removed and diagnostic code of acute renal failure were introduced, and three triggers were applied only to adults (Figure 1).

### 2.4. Evaluation of Drug-Related ED Visit Trigger Tool

For the evaluation of the newly developed DrEDTT, we analysed ED visits over a 6-month period from January 2016 to June 2016, after excluding ED visits due to traffic accidents and trauma, from four university hospitals in Korea with a combined annual ED census of approximately 150,000 visits.

We retrieved each hospital’s NEDIS data (chief complaints, diagnosis, and vital signs), ED discharge summary charts recorded by physician, data on medication use in the ED and laboratory data of the ED from the hospital’s electronic medical record (EMR) and transferred them into electronic case report forms (e-CRF). We included 25% of randomly selected ED visit cases to determine the sensitivity and specificity of DrEDTT after applying the query of DrEDTT to retrieved e-CRF data. In-depth chart review by two researchers (a pharmacist and a physician) at each hospital was performed and a true ADE was determined through causality assessment. Discrepancies between the findings of the two reviewers were resolved by consensus.

Categorical variables were expressed as a proportion of percentage (%). The positive predictive value (PPV) was determined as the number of cases in which an ADE was confirmed divided by the number of cases flagged by the trigger. We presented the PPV for each trigger with 95% confidence intervals. Sensitivity was calculated as the number of trigger-flagged ED visits determined as ADE-related by in-depth chart review divided by the total number of ADE-related ED visits identified by in-depth chart review. Specificity was calculated as unflagged ED visits divided by the number of ED visit that were determined as ADE-unrelated by chart review. Data management and statistical analysis were performed using SAS 9.3 (SAS Institute, Inc., Cary, NC, USA).

## 3. Results

### 3.1. Development of DrEDTT

Based on a literature review and expert group discussion, the final 28 triggers belonging to five categories were developed for detecting drug-related ED visits (Table 1). Symptom, vital sign, and laboratory data categories referred to the records of chief complaints and objective findings on ED records. Medication triggers referred to the specific medication order issued in the ED and identified cases which used medications for ADE symptom control, correcting abnormal signs, and as antidotes for overdose. The diagnostic code trigger consisted of 265 diagnosis codes describing ‘induced by medication’ or ‘poisoning by medication’ in reference to results from a systematic review by Hohl et al. [23]. The diagnostic code referred to the main diagnosis on the discharge summary.

### 3.2. Performance of DrEDTT in Multi-Center Cohort

There were differences among hospitals in terms of sex, age, and the purpose of the ED visit (Table S1). In this analysis, records of 66,564 patients were included after excluding ED visits due to traffic accidents (N = 3700) and trauma (N = 6460) by patients who visited the ED at four university hospitals from January 2016 to June 2016 (N = 76,724). At least one trigger was found in 21,268 (32.0%) patients. We randomly selected 25% of trigger-flagged (N = 5317) and unflagged ED visits (N = 11,324).

In total (N = 66,564) and sample population (N = 16,641), women accounted for 51.2% and 51.1% (*p* = 0.81), respectively. The mean age was 41.6 ± 25.9 and 41.5 ± 25.9 years (*p* = 0.78) and older adults aged 65 years or over made up 23.5% and 23.8% of the total and sample population, respectively (*p* = 0.80).

Among the randomly selected 25% sample, 959 true ADE cases (5.8%), comprising 745 trigger-flagged and 214 unflagged ED visits, were detected by chart reviews (Table 2).

The overall PPV associated with the DrEDTT was 14.0% (range for each individual trigger: 8.3% to 66.7%). The most frequently flagged trigger was the ‘antihistamine’ of medication trigger followed by the ‘anemia’ laboratory trigger, and ‘nausea or vomiting’ and ‘bleeding’ symptom triggers. The highest PPV was 66.7% for ‘3% sodium chloride’ followed by ‘INR > 4’ (60.0%), ‘blood glucose level < 50 mg/dL’ (50.0%), and ‘diagnosis codes for drug-induced disease’ (43.8%) (Table 3). The PPV of the composite diagnosis code category was highest at 35.3% (95% CI, 30.2–40.3%) followed by laboratory data category (18.4%, 95% CI, 17.0–19.9%) and medication category (16.0%; 95% CI, 14.5–17.4%).

The sensitivity and specificity of DrEDTT were 77.7% and 70.4%, respectively (Table 4). We found that the trigger-flagged ADEs included more preventable ADEs (17.3% vs. 11.2%, *p* < 0.001) and serious ADEs (35.2% vs. 20.6%, *p* < 0.001) than unflagged ADEs. The sensitivity of DrEDTT for identifying serious and preventable ADEs were 85.6% and 84.3%, respectively (Table 4).

## 4. Discussion

In this study, we developed a trigger tool to identify ADEs in patients visiting the ED for drug-related problems by taking advantage of the unique information available in ED medical records while considering the shortcomings of previous trigger tools. This newly developed trigger tool could be used in ADE surveillance systems for outpatients.

Karpov et al. evaluated the performance of existing trigger tools in identifying ADEs in adult patients presenting to the ED compared with those identified by the pharmacist’s point of care [8]. They concluded that reliance on trigger methods to detect ADEs is likely to be underestimated due to poor sensitivity (2.6% to 15.8%) and high PPV (57.1% to 100%). When we developed the DrEDTT, we intended to increase the sensitivity at the expense of a relatively low PPV to capture as many ADEs as possible. Therefore, the observed sensitivity, specificity, and PPV of DrEDTT were higher and lower than those of previously developed trigger tools [8].

The unique triggers included symptom triggers and diagnosis triggers, which helped us identify a broader range of ADEs, which might have partly contributed to the high sensitivity. For example, while ‘high INR values’ or ‘vitamin K administration’, included in most existing trigger tools were only able to capture bleeding by warfarin as an anticoagulant, ‘bleeding symptom’ or ‘intravenous PPI use’ included in the DrEDTT made it possible to identify bleeding-related ADEs caused by antiplatelet agents, non-steroidal anti-inflammatory drugs, and other novel anticoagulants. We developed a trigger tool that can detect predictable (type A) and non-predictable (type B) ADEs. In fact, “rash” analogue term in the symptom category, ‘chlorpheniramine, hydroxyzine’ in the medication category, and some diagnostic codes were intended to detect type B ADEs with the rest of the triggers targeting predictable ADEs.

From the 25% random sample of four hospital ED visits for reasons other than traffic accidents and trauma, we could estimate the prevalence of drug-related ED visits to be 5.8% of all-cause visits. Previous studies reported a wide range of prevalence of 0.4% to 28.1%. Various study factors such as detection methods and definition of ADEs as well as the study population might account for variations in the prevalence of ADEs leading to ED visits. Prospective studies [17,18,19,20,21,24] that detect ADEs at the point of care tend to estimate a higher incidence of ADEs, ranging from 0.8 to 28.1%, than do retrospective studies. A study by Tafreshi et al. [24], which was conducted in 253 patients over 35 days, reported the highest incidence of 28.1%. They estimated incidence after excluding 25% of total patients with difficulty in obtaining a good medication history, inability to communicate, and a shortage of available resources. On the other hand, another prospective observational study [15] reported the lowest incidence of 0.8%, which is significantly lower than the current study. They relied solely on the diagnosis of the physicians treating the patient visiting an ED. In contrast to inpatient care settings, ED physicians often do not consider ADEs to be the cause of ED visits due to a high workload, requirement of rapid diagnosis and treatment, and lack in comprehensive information such as detailed medication history [25].

The ‘diagnosis code for drug-induced disease’ trigger of the DrEDTT in this study, a concept similar to the physician-documented ADE, captured 77 ADE cases accounting for 0.5% of the total ED visits, which was surprisingly consistent with the result from a previous study that prospectively collected ED administrative data relying on physician diagnosis [4]. Based on these facts, concerns have been raised about the National Electronic Injury Surveillance System-Cooperative Adverse Drug Event Surveillance (NEISS-CADES), which mainly depends on the emergency physician’s case findings and documentation [26]. The prevalence of 5.8% estimated only by the trigger-based chart review in this study was higher than 0.4–4.2% reported in previous retrospective studies. Furthermore, in the recent study that applied IHI’s global trigger tool to the ED setting [27], the prevalence of adverse reactions in patients presenting to the ED was estimated at 2.3%, which is lower than that estimated by the DrEDTT developed in our study.

Although prospective surveillance methods involving clinical pharmacists for identifying ADE cases have been suggested and are ideal for identifying more ADEs leading to ED visits [28], their application would not feasible in most health care institutions due to a shortage of pharmacists and high costs [26]. As an alternative, we suggest that trigger-based chart review be used considering that its efficiency is excellent [27], minimal training is required for use, it is a population-specific application, and it allows for a sampling strategy. The DrEDTT developed in this study could be used to expand NEDIS to develop a nation-wide outpatient ADE surveillance system. More specifically, DrEDTT could be applied as automated ADE detection in the EMR for patients visiting the ED to help real-time ADE detection, allowing for timely ED interventions.

To the best of our knowledge, this was the first trigger tool designed specifically for detecting ED visits related to ADEs. However, a few limitations should be considered. First, the sensitivity results might have been overestimated because we considered true ADEs as total ADEs determined by in-depth chart review with the limitation of a retrospective study design. Although there were no globally agreed-upon standard methods for identifying ADEs, prospectively well-designed surveillance might help increase ADE identification. Therefore, validation of this trigger tool with a prospectively designed study is warranted. Second, due to the nature of the retrospective study, the incidence of drug-related ED visits might have been underestimated. However, the retrospective design is not always inferior. As shown in the study of Wolff and Bourke [29], retrospective reviews involving a careful consideration of the facts allow determination of ADEs that could not have been identified at the point of care, especially, if the case was flagged by the trigger. Additional information that could be obtained during the follow-up period could be available for a trigger targeted chart review. Third, when applying this trigger tool, it should be kept in mind that drug-induced delirium and falls, one of major causes of emergency room visits especially in the elderly [30,31], could not be caught with this trigger tool. DrEDTT could not include some type of ADE if there are no specific laboratory data or specific medication use or diagnostic code implicating them. However, this might also have a limitation in most retrospective study designs because physicians often fail to recognize the medications as the cause of falls and delirium in the ED environment. Fourth, this newly developed trigger tool has been applied in four university hospitals in Korea. Generalizability to other types of emergency medical services and other countries with different healthcare systems needs to be investigated further.

## 5. Conclusions

In this study, a set of triggers specifically designed for detecting drug-related ED visits was developed, the sensitivity and specificity of which were 77.7% and 70.4%, respectively. This newly developed trigger tool might assist in real-time automatic ADE detection allowing timely interventions for patients visiting the ED, and can be extended to the surveillance of outpatient ADEs.

## Figures and Tables

**Table 1:** The final drug-related emergency department visit trigger tool (DrEDTT) list.

Category	Trigger	
Symptom	T1	“rash” analogue term
	T2	nausea or vomiting
	T3	“bleeding” analogue term
	T4	palpitation
Vital sign	T5	systolic blood pressure < 80 mmHg
	T6	systolic blood pressure > 180 mmHg or diastolic blood pressure >110 mmHg
	T7	heart rate (HR) < 50
Laboratory data	T8	International Normalized Ratio (INR) > 4
	T9	blood glucose level < 50 mg/dL
	T10	blood glucose level > 300 mg/dL
	T11	white blood cell < 3000/mm^3^ or absolute neutrophil count < 1500/mm^3^
	T12	platelet < 50,000/mm^3^
	T13	hemoglobin < 10 g/dL
	T14	alanine aminotransferase (ALT) > 84 U/L
	T15	serum sodium < 130 mEq/L
	T16	serum potassium > 6.0 mEq/L
Medication	T17	chlorpheniramine, hydroxyzine
	T18	serotonin(5-HT3) antagonist
	T19	vitamin K
	T20	intravenous proton pump inhibitors
	T21	10% dextrose, 50% dextrose
	T22	loperamide, smectite, rifaximin
	T23	flumazenil without concomitant midazolam
	T24	dimenhydrinate
	T25	3% sodium chloride
	T26	polystyrene sulfonate calcium
Diagnosis code	T27	diagnosis codes of acute renal failure
	T28	diagnosis codes for drug induced disease

**Table 2:** Determination of the prevalence of drug-related ED visits by using DrEDTT in a multi-center study.

	Total	A Hospital	B Hospital	C Hospital	D Hospital
ED visits	66,564	16,280	18,718	18,420	13,146
Trigger flagged ED visits, N (% of ED visits)	21,268 (32.0%)	4650 (28.6%)	4441 (23.7%)	6597 (35.8%)	5580 (42.4%)
25% sampled ED visits	16,641	4067	4678	4635	3261
ADE cases, N (prevalence, %)	959 (5.8%)	333 (8.2%)	211 (4.5%)	191 (4.1%)	224 (6.9%)

ED, Emergency Department; ADEs, Adverse Drug Events; DrEDTT, drug-related ED visit trigger tool.

**Table 3:** Positive predictive value of 28 individual drug-related ED visit triggers in a multi-center study.

Trigger		Trigger Flagged Cases (N)	PPV (%, 95% CI)	
T1	“rash” analogue term	452	20.8%	(20.2–21.4%)
T2	nausea or vomiting *	632	10.1%	(9.7–10.6%)
T3	“bleeding” analogue term	572	17.1%	(16.6–17.7%)
T4	palpitation	118	13.6%	(13.0–14.1%)
T5	SBP < 80 mmHg	125	13.6%	(13.1–14.1%)
T6	SBP > 180mmHg or DBP > 110 mmHg	524	12.4%	(11.9–12.9%)
T7	heart rate (HR) < 50	53	22.6%	(22.0–23.3%)
T8	INR > 4	10	60.0%	(59.3–60.7%)
T9	blood glucose level < 50 mg/dL	46	50.0%	(49.2–50.8%)
T10	blood glucose level > 300 mg/dL	292	18.2%	(17.6–18.7%)
T11	WBC < 3000/mm^3^ or ANC < 1500/mm^3^	273	27.1%	(26.4–27.8%)
T12	platelet < 50,000/mm^3^	134	20.9%	(20.3–21.5%)
T13	hemoglobin < 10 g/dL	1061	16.0%	(15.5–16.6%)
T14	ALT > 84 U/L	487	12.1%	(11.6–12.6%)
T15	serum sodium < 130 mEq/L	410	19.0%	(18.4–19.6%)
T16	serum potassium > 6.0 mEq/L	97	32.0%	(31.3–32.7%)
T17	chlorpheniramine, hydroxyzine	1175	13.2%	(12.7–13.7%)
T18	serotonin(5-HT3) antagonist *	112	13.4%	(12.9–13.9%)
T19	vitamin K	27	25.9%	(25.3–26.6%)
T20	intravenous proton pump inhibitors	378	14.8%	(14.3–15.4%)
T21	10% dextrose, 50% dextrose	335	27.2%	(26.5–27.8%)
T22	loperamide, smectite, rifaximin *	143	13.3%	(12.8–13.8%)
T23	flumazenil without concomitant midazolam	17	29.4%	(28.7–30.1%)
T24	dimenhydrinate	120	8.3%	(7.9–8.8%)
T25	3% sodium chloride	6	66.7%	(66.0–67.4%)
T26	polystyrene sulfonate calcium	60	28.3%	(27.6–29.0%)
T27	diagnoses codes of acute renal failure	167	26.3%	(25.7–27.0%)
T28	diagnoses codes for drug induced disease	176	43.8%	(43.0–44.5%)
Total		5317	14.0%	(13.5–14.5%)

CI, confidence intervals; SBP, Systolic Blood Pressure; DBP, Diastolic Blood Pressure; WBC, White Blood Cell; ANC, Absolute Neutrophil Count; ALT, alanine aminotransferase; INR, International Normalized Ratio. * T2, T18, and T22 were applied only to adult (18 years and older).

**Table 4:** Sensitivities and Specificities of DrEDTT for detecting total, serious, and preventable ADE cases.

a	Trigger Flagged ED Visits(N = 5317)	Trigger Unflagged ED Visits(N = 11,110)	Sensitivity	Specificity
Total ADE cases (N = 959)	745	214	77.7%	70.4%
Serious ADE cases (N = 306)	262	44	85.6%	68.6%
Preventable ADE cases (N = 153)	129	24	84.3%	68.1%

ED, emergency department; ADEs, adverse drug events; DrEDTT, drug-related ED visit trigger tool.

## Data Availability

The data presented in this study are available on request from the corresponding author.

## Supplementary Materials

The following are available online at https://www.mdpi.com/article/10.3390/ijerph18168572/s1, Table S1: The basic characteristics of patients who visits ED in the each hospital during the study period.
