# Supplementary material for: Development and Validation of a Trigger Tool for Identifying Drug-Related Emergency Department Visits
Source: Int J Environ Res Public Health. 2021 Aug 13;18(16):8572. doi: 10.3390/ijerph18168572 (PMC8391800; doi:10.3390/ijerph18168572)
Supplement: Supplementary file 1 [file ijerph-18-08572-s001.zip › ijerph-1308643-supplementary.pdf]

Supplemental Table S1. The basic characteristics of patients who visits ED in the each hospital during the study period

		A hospital	B hospital	C hospital	D hospital	Total	*P-value*
All cause ED visits	N	18,804	22,627	20,607	14,686	76,724	
Traffic accidents	N (%)	1,013 (5.4%)	1,066 (4.7%)	791 (3.8%)	830 (5.7%)	3,700 (4.8%)	
Trauma		1,511 (8.0%)	2,843 (12.6%)	1,396 (6.8%)	710 (4.8%)	6,460 (8.4%)	
Sex (Female)	N (%)	9,571 (50.9%)	11,879 (52.5%)	9,788 (47.5%)	6,594 (44.9%)	37,833 (44.9%)	<0.001
Age		38.2±23.7	35.6±25.5	41.2±26.6	51.2±23.3	40.7±25.6	<0.001
0 - 17	N (%)	1,918 (10.2%)	2,919 (12.9%)	2,782 (13.5%)	911 (6.2%)	8,529 (11.1%)	
18 - 64	N (%)	13,426 (71.4%)	15,251 (67.4%)	12,570 (61.0%)	8,577 (58.4%)	49,824 (64.9%)	
≥ 65	N (%)	3,460 (18.4%)	4,458 (19.7%)	5,255 (25.5%)	5,199 (35.4%)	18,371 (24.0%)	

ED, Emergency Department
